# Key therapeutic targets implicated at the early stage of hepatocellular carcinoma identified through machine-learning approaches

**DOI:** 10.1038/s41598-023-30720-x

**Published:** 2023-03-07

**Authors:** Seyed Mahdi Hosseiniyan Khatibi, Farima Najjarian, Hamed Homaei Rad, Mohammadreza Ardalan, Mohammad Teshnehlab, Sepideh Zununi Vahed, Saeed Pirmoradi

**Affiliations:** 1 Kidney Research Center, Tabriz University of Medical Sciences, Daneshgah Street, Tabriz, 51665118 Iran; 2 Clinical Research Development Unit of Tabriz Valiasr Hospital, Tabriz University of Medical Sciences, Niyayesh Blvd., Tabriz, Iran; 3 Rahat Breath and Sleep Research Center, Tabriz University of Medical Science, Tabriz, Iran; 4 Faculty of Medicine, Tabriz University of Medical Sciences, Tabriz, Iran; 5 Department of Electric and Computer Engineering, K.N. Toosi University of Technology, Tehran, Iran

**Keywords:** Cancer genomics, Machine learning

## Abstract

Hepatocellular carcinoma (HCC) is the most frequent type of primary liver cancer. Early-stage detection plays an essential role in making treatment decisions and identifying dominant molecular mechanisms. We utilized machine learning algorithms to find significant mRNAs and microRNAs (miRNAs) at the early and late stages of HCC. First, pre-processing approaches, including organization, nested cross-validation, cleaning, and normalization were applied. Next, the t-test/ANOVA methods and binary particle swarm optimization were used as a filter and wrapper method in the feature selection step, respectively. Then, classifiers, based on machine learning and deep learning algorithms were utilized to evaluate the discrimination power of selected features (mRNAs and miRNAs) in the classification step. Finally, the association rule mining algorithm was applied to selected features for identifying key mRNAs and miRNAs that can help decode dominant molecular mechanisms in HCC stages. The applied methods could identify key genes associated with the early (e.g., Vitronectin, thrombin-activatable fibrinolysis inhibitor, lactate dehydrogenase D (LDHD), miR-590) and late-stage (e.g., SPRY domain containing 4, regucalcin, miR-3199-1, miR-194-2, miR-4999) of HCC. This research could establish a clear picture of putative candidate genes, which could be the main actors at the early and late stages of HCC.

## Introduction

Hepatocellular carcinoma (HCC) is the third cause of cancer deaths worldwide^1^. The scientific observations have indicated that cirrhosis^2^, heavy alcoholism^3^, smoking, lifestyle, hepatitis B and C viral infection^4,5^, hemochromatosis^6^, and alpha-1-antitrypsin deficiency can be important HCC risk factors. Liver function, clinical expertise, availability of treatment resources, and cancer stage can affect treatment procedures. Due to the diagnosis of liver cancer at the late stages, the overall survival rate of HCC patients has not increased despite advancements in treatment^7^. HCC early-stage detection allows clinicians to use a wide range of treatments^8^, playing an essential role in making treatment decisions. Moreover, the identification of dominant molecular mechanisms at the early and late stages can improve treatment strategies.

Traditionally, clinicians utilized alpha-fetoprotein (AFP) and AFP-L3 (a glycoform of AFP) as HCC biomarkers in most developing countries^7,9^; however, these biomarkers have no reliability, sufficient sensitivity, and specificity^8^. Another biomarker was Des-gamma-carboxyprothrombin (DCP), which is upregulated at late stages^10–12^. In recent years, next-generation sequencing (NGS) technology and bioinformatics methods have provided promising ways for the identification of biomarkers^13^. Many studies detected the expression of cancer-associated genes and indicated their vital role in hepatocarcinogenesis. Previous studies aimed to identify the differentially expressed RNA transcripts, genes, or miRNAs in cancer versus normal or cancer versus other liver diseases.

Recently, Artificial Intelligence has succeeded in many applications, such as health care^14^. In this regard, a few studies proposed machine learning methods to predict the HCC stages based on the genomic profile of samples^15^. Sathipati et al. suggested a support vector machine-cancer stage prediction method and a bi-objective genetic algorithm for miRNA selection. Test accuracy and AUC (area under the receiver operating characteristic curve) of 74.28%, and 0.73 for early (stage I, II) and late (stage III, IV) stages of discrimination were reported based on miRNA data, respectively^16^. Kaur et al. investigate mRNA and methylation data to distinct early (stage I) and late (stage II, III, IV) stages. They utilized different feature selection and classification algorithms and compared the obtained result. Accuracy and AUC of 76% and 0.79 were reported, respectively^17^. In these studies, authors applied hold-out cross-validation for error estimation with an 80:20 ratio for training and test splitting. Moreover, a machine learning approach was applied to the early diagnosis of HCC and classifying patients with HCC and without HCC (CwoHCC)^18^. In another study, authors utilized machine learning and bioinformatic tools to diagnose HCC patients (HCC and non-HCC)^19^. Książek et al.^20^ used a two-level feature selection method (NCA-GA-SVM) for HCC fatality prognosis prediction. Recently, Liu et al.^21^ proposed a deep-learning model to predict HCC recurrence based on pathology images.

In this study, we applied machine learning algorithms to investigate significant mRNAs and miRNAs separately. First, we applied pre-processing approaches, including organization, nested cross-validation, cleaning, and normalization. Next, the t-test/ANOVA methods and binary particle swarm optimization (PSO) were used as a filter and wrapper method in the feature selection step, respectively. Then, a classifier based on machine learning and deep learning algorithms was utilized to evaluate the selected features (mRNAs and miRNAs) in the classification step. Finally, the association rule mining algorithm was applied to selected features for identifying key mRNAs and miRNAs that can help decode dominant molecular mechanisms at the early and late stages of HCC.

## Results

**Figure 1:**
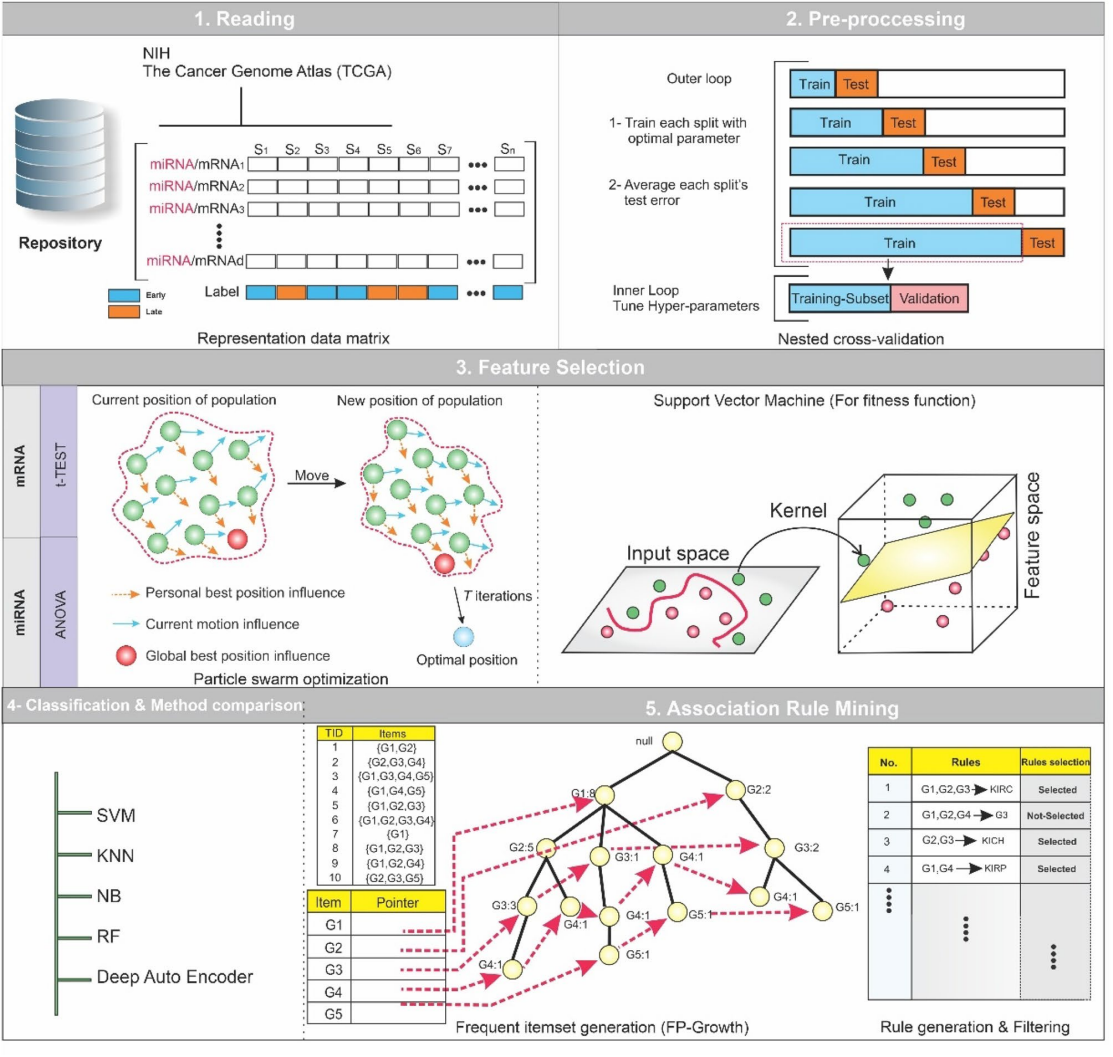
The overview of the proposed method. Five main steps were applied to miRNA and mRNA expression data separately, including reading, preprocessing, feature selection, classification, and association rule mining. (1) In the reading step, each dataset was downloaded from the TCGA repository. (2) The preprocessing step includes two sub-steps, nested cross-validation, and normalization. (3) The feature selection step contains two sub-steps: the filter method based on t-test for mRNA data and ANOVA for miRNA data, and the wrapper method based on binary particle swarm optimization (PSO) for both mRNA and miRNA data, in which candidate miRNAs/mRNAs with more relevance to early-stage and late-stage Hepatocellular Carcinoma (HCC) were selected. (4) multiclassifier models were utilized to evaluate the discrimination power of selected miRNAs/mRNAs. (5) The Association Rule Mining method discovered the hidden relationship between selected miRNAs/mRNAs at the early-stage and late-stage of HCC in the first level and the complex relationship among selected miRNAs/mRNAs in the second level.

Our primary objective was to identify the significant mRNAs/miRNAs that can classify patients at early-stage and late-stage with the best accuracy in the first phase. Decoding the molecular mechanisms of early- and late-stages and identifying their top mRNAs/miRNAs were our next objectives. In this regard, we applied four steps to mRNA/miRNA data, including preprocessing, feature selection, classification, and association rule mining as shown in Fig. 1. Moreover, we used Python and its libraries, including Numpy, Pandas, Matplotlib, Sickit-learn, Scipy, Pytorch, Pyswarms, and Mlxtend to implement the proposed algorithms.

In the feature selection step, t-test (filter method) and binary PSO (wrapper method) were used for selecting significant mRNAs and miRNAs. Finally, 77 miRNAs among 1881 miRNAs were selected, presented in Table S1. Furthermore, 123 mRNAs among 60,483 mRNAs were selected (Table S2). The binary PSO parameters, including α, β, θ, number of particles, and number of iterations, were set to 2, 2, 0.9, 35, and 100, respectively.

In the classification step, we employed seven classifiers, including SVM, KNN, NB, RF, deep Self-Organizing Auto-Encoder (SOAE), Logistic Regression, and XgBoost to evaluate the importance of selected features (mRNAs/miRNAs) based on their discrimination power between early and late stages. The average performance of each classifier was represented using accuracy, F1-score, MCC, sensitivity, and specificity for train/validation/test folds of miRNA and mRNA data in Tables 1 and 2, respectively. Moreover, we reported the performance of classifiers based on both selected mRNAs and miRNAs by concatenating chosen features (Table 3).

**Table 1:** The performance of classifiers based on 77 selected miRNAs.

Classifier	Folds	Accuracy	AUC-ROC	F1-score (Early stage)	F1-score (Late stage)	MCC	Sn	Sp
SVM	Train	88.8	0.88	0.89	0.88	0.78	0.81	0.95
	Validation	71.3	0.71	0.72	0.7	0.43	0.67	0.75
	Test	**70**	**0.7**	**0.7**	**0.68**	**0.4**	**0.68**	**0.73**
KNN	Train	72.2	0.72	0.75	0.67	0.47	0.56	0.87
	Validation	56.2	0.56	0.62	0.46	0.14	0.39	0.74
	Test	57.1	0.56	0.63	0.45	0.14	0.37	0.75
NB	Train	70	0.7	0.74	0.63	0.43	0.51	0.89
	Validation	66.1	0.66	0.7	0.59	0.34	0.5	0.81
	Test	66.3	0.65	0.67	0.59	0.32	0.53	0.78
RF	Train	91.4	0.91	0.91	0.9	0.83	0.85	0.97
	Validation	65	0.65	0.67	0.61	0.3	0.59	0.74
	Test	65	0.66	0.67	0.61	0.32	0.58	0.74
AE	Train	75	0.74	0.75	0.74	0.5	0.71	0.78
	Validation	66	0.66	0.67	0.64	0.33	0.62	0.7
	Test	65.1	0.65	0.66	0.62	0.3	0.6	0.7
Logistic regression	Train	82.7	0.82	0.82	0.82	0.65	0.8	0.84
	Validation	62.7	0.63	0.63	0.61	0.26	0.6	0.65
	Test	62.3	0.62	0.62	0.6	0.24	0.6	0.64
XgBoost	Train	98.5	0.98	0.98	0.98	0.97	0.97	0.99
	Validation	64.2	0.64	0.64	0.63	0.29	0.61	0.67
	Test	63	0.63	0.63	0.6	0.26	0.59	0.66

Significant values are in bold. AUC: The area under the curve, ROC: receiver operating characteristic curve, MCC: Matthews Correlation Coefficient, Sn: sensitivity, Sp: specificity.

**Table 2:** The performance of classifiers based on 123 selected mRNAs.

Classifier	Folds	Accuracy	AUC-ROC	F1-score (Early stage)	F1-score (Late stage)	MCC	Sn	Sp
SVM	Train	93	0.93	0.93	0.93	0.86	0.95	0.91
	Validation	77.6	0.77	0.77	0.77	0.55	0.74	0.8
	Test	**74.7**	**0.75**	**0.74**	**0.74**	**0.5**	**0.73**	**0.76**
KNN	Train	70	0.7	0.6	0.76	0.46	0.94	0.46
	Validation	60	0.6	0.44	0.7	0.36	0.9	0.31
	Test	59	0.58	0.41	0.67	0.2	0.85	0.31
NB	Train	79	0.79	0.79	0.78	0.58	0.77	0.81
	Validation	70	0.7	0.72	0.68	0.42	0.64	0.77
	Test	68	0.7	0.7	0.62	0.38	0.59	0.78
RF	Train	92.3	0.92	0.92	0.92	0.84	0.89	0.95
	Validation	65.3	0.65	0.66	0.64	0.31	0.62	0.68
	Test	63.6	0.64	0.63	0.63	0.28	0.6	0.68
AE	Train	79.5	0.79	0.79	0.79	0.59	0.78	0.8
	Validation	71.4	0.71	0.71	0.71	0.43	0.7	0.72
	Test	70	0.71	0.7	0.7	0.42	0.68	0.73
Logistic regression	Train	95.2	0.95	0.95	0.95	0.9	0.95	0.94
	Validation	68.7	0.68	0.68	0.68	0.37	0.68	0.68
	Test	67.7	0.68	0.65	0.69	0.36	0.71	0.64
XgBoost	Train	92.4	0.92	0.92	0.92	0.85	0.89	0.94
	Validation	60	0.6	0.6	0.58	0.2	0.57	0.62
	Test	61.2	0.61	0.59	0.61	0.23	0.62	0.61

Significant values are in bold. AUC: The area under the curve, ROC: receiver operating characteristic curve, MCC: Matthews Correlation Coefficient, Sn: sensitivity, Sp: specificity.

**Table 3:** The performance of classifiers based on 200 selected mRNAs and miRNAs.

Classifier	Folds	Accuracy	AUC-ROC	F1-score (Early stage)	F1-score (Late stage)	MCC	Sn	Sp
SVM	Train	96.1	0.96	0.96	0.96	0.92	0.94	0.97
	Validation	75.4	0.75	0.76	0.74	0.51	0.7	0.8
	Test	**76.9**	**0.77**	**0.78**	**0.74**	**0.54**	**0.7**	**0.83**
KNN	Train	76.2	0.76	0.72	0.79	0.54	0.88	0.63
	Validation	64	0.64	0.59	0.68	0.29	0.79	0.48
	Test	63.2	0.64	0.56	0.67	0.29	0.8	0.47
NB	Train	78.7	0.76	0.8	0.72	0.56	0.59	0.93
	Validation	68	0.67	0.72	0.61	0.38	0.5	0.85
	Test	67.7	0.67	0.63	0.64	0.36	0.64	0.71
RF	Train	94	0.94	0.94	0.93	0.83	0.9	0.97
	Validation	67.8	0.68	0.7	0.64	0.36	0.6	0.76
	Test	69.5	0.69	0.71	0.66	0.4	0.63	0.75
AE	Train	82	0.81	0.82	0.81	0.64	0.8	0.84
	Validation	74	0.74	0.74	0.73	0.48	0.72	0.76
	Test	75	0.74	0.76	0.72	0.5	0.7	0.79
Logistic regression	Train	100	1	1	1	1	1	1
	Validation	71	0.7	0.7	0.7	0.41	0.71	0.7
	Test	74.7	0.74	0.75	0.73	0.49	0.75	0.74
XgBoost	Train	95	0.95	0.95	0.94	0.9	0.91	0.97
	Validation	64.6	0.65	0.65	0.62	0.3	0.6	0.69
	Test	63	0.63	0.64	0.6	0.26	0.6	0.65

Significant values are in bold. AUC: The area under the curve, ROC: receiver operating characteristic curve, MCC: Matthews Correlation Coefficient, Sn: sensitivity, Sp: specificity.

The performance of classifiers based on miRNA features illustrated that SVM with 70% accuracy and 0.7 AUC was the best model. SVM was also the best classifier in mRNA features with 74.7% accuracy and 0.75 AUC. In addition, concatenating mRNAs and miRNAs improved the classification performance with an accuracy of 76.9 and an AUC of 0.77. Also, all measures were calculated based on the nCV, which is the most accurate error estimation approach in the real world. In the association rule mining step, we discovered interesting relations including feature(s)-feature(s) (mRNAs/miRNAs) and feature(s)-target (early-stage/late-stage). Moreover, we selected significant mRNAs/miRNAs based on the repeat count of these features in generated rules and studied their role in the early and late stages of HCC tumors.

### miRNA data association rule mining analysis

Twenty-eight top miRNAs involved in the consequence of early-stage and late-stage rules were presented based on the repeat counts in Table 4. In miRNA data, parameters of the algorithm, including lift (association rule), max-length (maximum length of frequent itemset), and min-support (frequent itemset) were set to 1.1, 4, and 0.3, respectively. Also, twenty of the top early-stage and late-stage rules were presented as the if–then form in Supplementary Tables S3 and S4, respectively.

**Table 4:** Top miRNAs and mRNAs based on repeat count in early-stage and late-stage rules.

Early-stage rules		Late-stage rules	
miRNA ID	Repeat count	miRNA ID	Repeat count
hsa-mir-590	1330	hsa-mir-3199-1	351
hsa-mir-23a	827	hsa-mir-194-2	168
hsa-mir-4443	662	hsa-mir-4999	108
hsa-mir-3691	448	hsa-mir-885	85
hsa-mir-877	447	hsa-mir-151b	70
hsa-mir-331	427	hsa-mir-4654	52
hsa-mir-6515	396	hsa-mir-216b	38
hsa-mir-629	376	hsa-mir-22	37
hsa-mir-4764	355	hsa-mir-126	33
hsa-mir-7850	273	hsa-mir-3926-1	19
hsa-let-7e	256	hsa-mir-4526	18
hsa-mir-4523	238	hsa-mir-330	17
hsa-mir-1289-1	213	hsa-mir-641	17
hsa-mir-1255a	212	hsa-mir-3622a	15
hsa-mir-6888	211	hsa-mir-4673	13
hsa-mir-6801	206	hsa-mir-6845	13
hsa-mir-4752	206	hsa-mir-548v	12
hsa-mir-4487	192	hsa-mir-6728	12
hsa-mir-5706	179	hsa-mir-3155a	12
hsa-mir-95	171	hsa-mir-3936	11
hsa-mir-423	166	hsa-mir-6783	10
hsa-mir-4746	165	hsa-mir-4735	10
hsa-mir-183	158	hsa-mir-548s	9
hsa-mir-1254-2	145	hsa-mir-3680-1	9
hsa-mir-643	141	hsa-mir-3926-2	9
hsa-mir-561	133	hsa-mir-1257	9
hsa-mir-4478	127	hsa-mir-4757	9
hsa-mir-658	121	hsa-mir-124-1	9

mRNA ID	Repeat count	mRNA ID	Repeat count
ENSG00000109072.12	1553	ENSG00000176422.12	7297
ENSG00000080618.12	1398	ENSG00000130988.11	7086
ENSG00000166816.12	1039	ENSG00000080618.12	6969
ENSG00000137806.7	931	ENSG00000109072.12	6949
ENSG00000146416.15	254	ENSG00000166816.12	6871
ENSG00000130307.10	254	ENSG00000137806.7	6668
ENSG00000255987.1	250	ENSG00000055957.9	6661
ENSG00000245954.5	250	ENSG00000036473.6	6372
ENSG00000245164.5	250	ENSG00000167711.12	6355
ENSG00000236213.1	250	ENSG00000125730.15	6042
ENSG00000233387.1	250	ENSG00000146416.15	5915
ENSG00000211751.6	250	ENSG00000161944.15	5723
ENSG00000211749.1	250	ENSG00000121410.10	5706
ENSG00000246084.2	250	ENSG00000188338.13	5588
ENSG00000163815.5	250	ENSG00000163631.15	5531
ENSG00000237702.2	250	ENSG00000244414.5	5256
ENSG00000124203.5	247	ENSG00000147647.11	5243
ENSG00000113263.11	247	ENSG00000139597.15	5194
ENSG00000264468.1	247	ENSG00000167701.12	5162
ENSG00000010319.5	237	ENSG00000134240.10	5075
ENSG00000178343.4	233	ENSG00000185305.9	4933
ENSG00000273328.4	229	ENSG00000172482.4	4890
ENSG00000264419.1	225	ENSG00000178301.3	4460
ENSG00000197921.5	219	ENSG00000213995.10	4328
ENSG00000270412.1	216	ENSG00000157379.12	4248
ENSG00000174990.4	213	ENSG00000154734.13	4230
ENSG00000231690.2	213	ENSG00000173269.12	4134
ENSG00000272789.1	204	ENSG00000170989.8	3992

In Supplementary Fig. S1a,b, the Spearman correlation for five top miRNAs of early-stage and late-stage was shown based on the heatmap plot, respectively. Also, the strength distribution of early-stage and late-stage association rules according to their lift, support, and confidence was shown in Supplementary Fig. S1c,d, respectively. We displayed the repeat count of 28 top miRNAs as ring bar plots in Supplementary Fig. S1e,f for early-stage and late-stage rules, respectively. Moreover, the boxplots of the top three miRNAs with a high repeat count at the early-stage and late-stage association rules were shown in Supplementary Fig. S2a,b, respectively.

**Figure 2:**
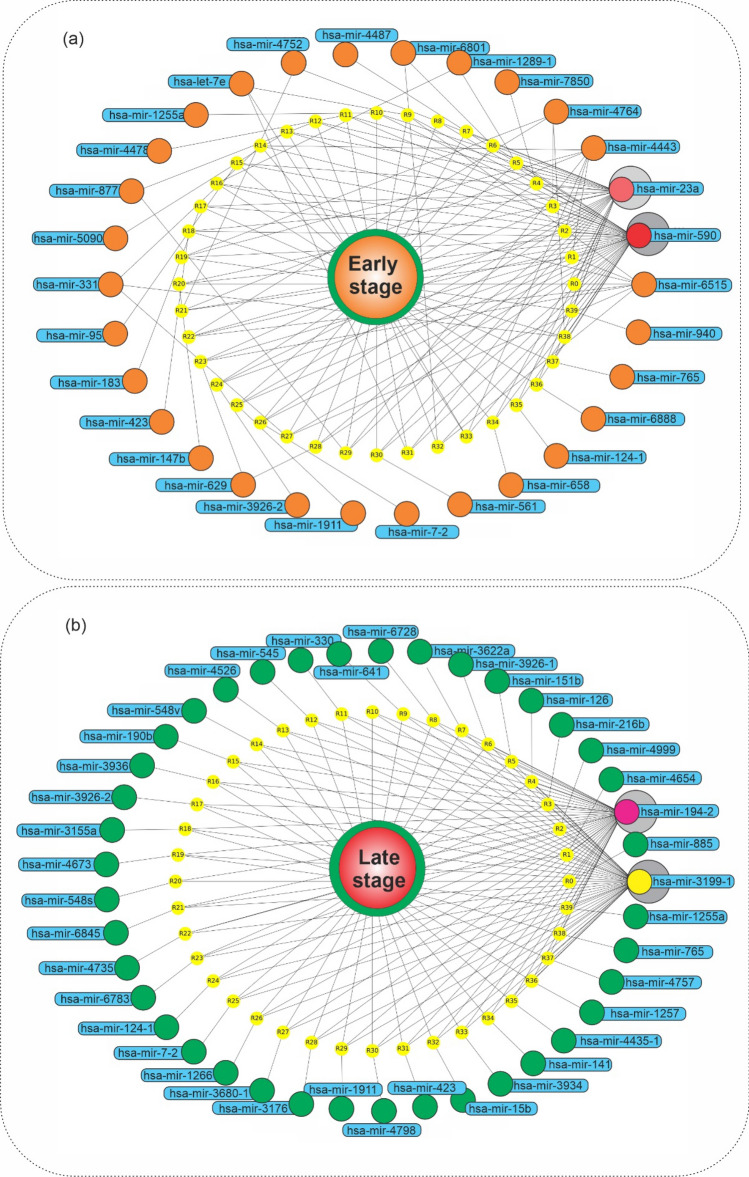
Graph network of miRNAs at early- and late-stage of HCC. Graph network of (**a**) early-stage related association rules (with lift > 1.16) and (**b**) late-stage related association rules (with lift > 1.2), in which the early-stage phenotype, its rules, and related miRNAs were presented, by orange, yellow, and blue colors, respectively. Python programming language (version 3.9) and Matplotlib library (version 3.6.0) were used to draw the plots, all of them are open sources.

In addition, we showed features-phenotype associations according to association rules in the graph network (Fig. 2). In Fig. 2a, it is obvious that early-stage phenotype, based on early-stage association rules, is highly dependent on miR-590, miR-23a, miR-4443, and miR-4764. In Fig. 2b, it is obvious that the late-stage phenotype is dependent on miR-3199-1 and miR-194.2.

**Figure 3:**
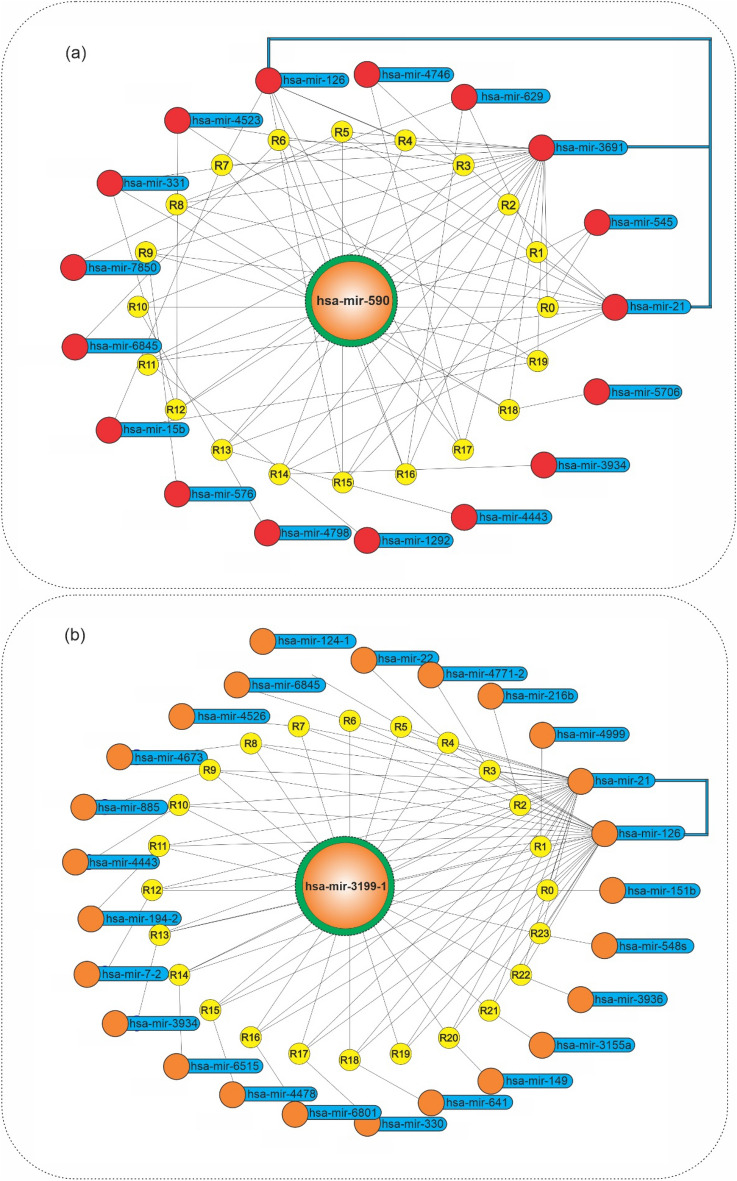
Graph network of has-mir-590 and has-mir-3199-1 in HCC. Graph network of (**a**) has-mir-590 (with lift > 1.14) and (**b**) has-mir-3199-1 (with lift > 1.126) related association rules, in which the has-mir-590 and has-mir-3199-1, their rules, and their related miRNAs were presented, by orange, yellow, and blue colors, respectively. Python programming language (version 3.9) and Matplotlib library (version 3.6.0) were used to draw the plots, all of them are open sources.

The hsa-mir-590 was the most frequent itemset in early-stage association rules (1330 repeat counts). Therefore, this miRNA was investigated based on association rules in the graph network to find its relation with other miRNAs (Fig. 3). As shown in Fig. 3a, it is obvious that the most frequent miRNA (miR-590) at the early-stage association rules is associated with miR-3691, miR-21, and miR-126. The hsa-mir-3199-1 was the most frequent itemset in late-stage association rules (with 351 repeat counts) that has a high dependency on miR-21 and miR-126 (Fig. 3b).

### mRNA data association rule mining analysis

Twenty-eight top mRNAs involved in the consequence of early-stage and late-stage rules were presented based on the repeat counts in Table 4. In mRNA data, parameters of the algorithm, including lift, max-length, and min-support were set to 1.1, 4, and 0.2, respectively. Moreover, twenty of the top early-stage and late-stage rules were presented as the if–then form in Supplementary Tables S5 and S6, respectively.

In Supplementary Fig. S3a,b, the Spearman correlation for five top mRNAs of early-stage and late-stage was shown based on the heatmap plot, respectively. The strength distribution of early-stage and late-stage association rules in line with their support, lift, and confidence was demonstrated in Supplementary Fig. S3c,d, respectively. We displayed the repeat count of 28 top mRNAs as ring bar plots in Supplementary Fig. S3e,f for early-stage and late-stage rules. Also, the boxplots of three top mRNAs with a high repeat count at the early-stage and late-stage association rules were shown in Supplementary Fig. S2c,d, respectively.

**Figure 4:**
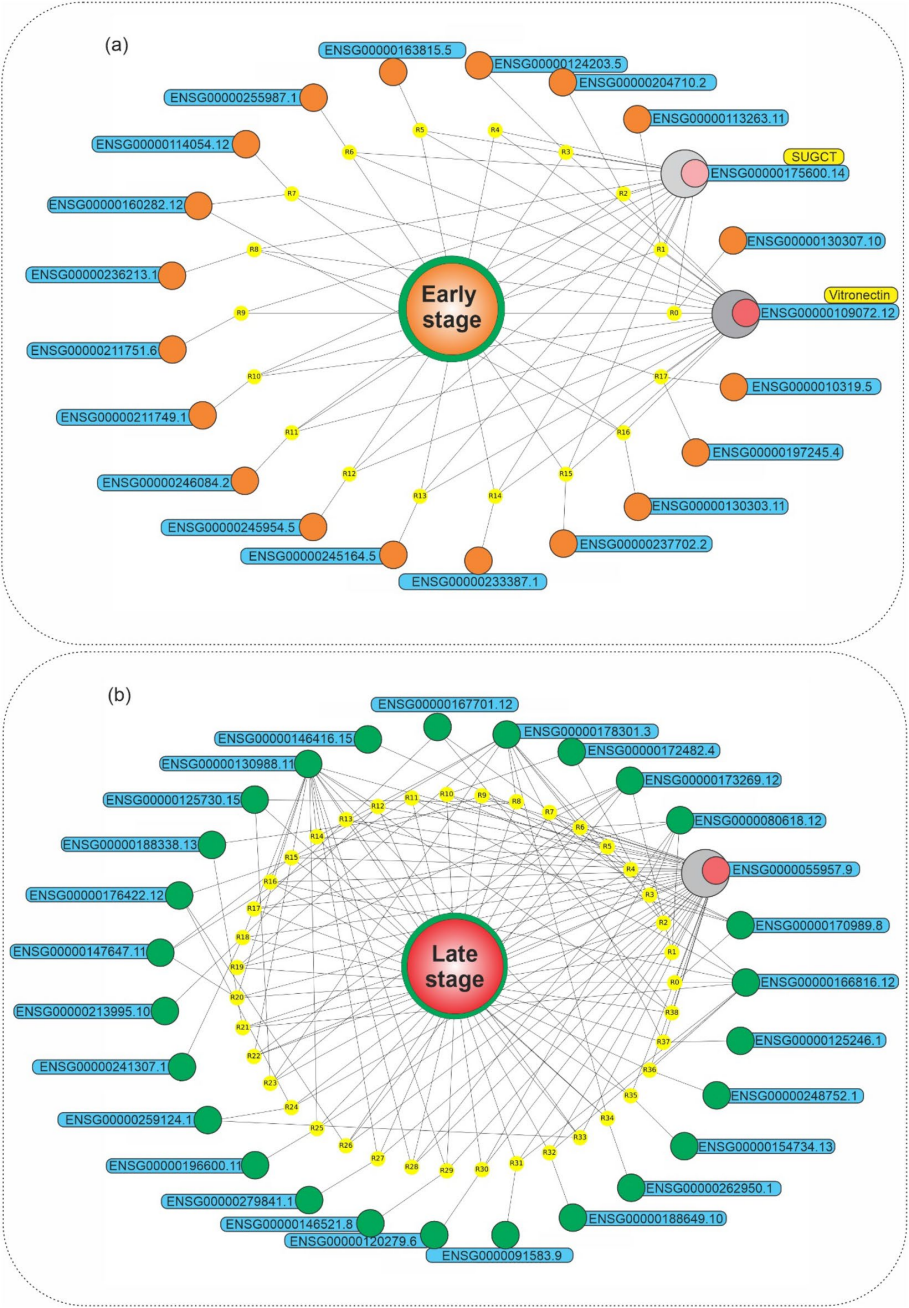
Graph network of mRNAs at early- and late-stages of HCC. Graph network of (**a**) early-stage related association rules (with lift > 1.21) and (**b**) late-stage related association rules (with lift > 1.38), in which the early-stage phenotype, its rules, and related mRNAs were presented, by orange, yellow, and blue colors, respectively. Python programming language (version 3.9) and Matplotlib library (version 3.6.0) were used to draw the plots, all of them are open sources.

In addition, we showed features-phenotype associations based on association rules in the graph network (Fig. 4). Early-stage phenotype had a high dependency on ENSG00000109072 (Vitronectin) and ENSG00000175600 (SUGCT, succinyl-CoA:glutarate-CoA transferase), Fig. 4a. In Fig. 4b, it is obvious that late-stage phenotype, based on late-stage association rules, is highly dependent on ENSG0000055957, ENSG00000178301, ENSG00000130988, ENSG00000173269, ENSG0000080618, and ENSG00000116816.

**Figure 5:**
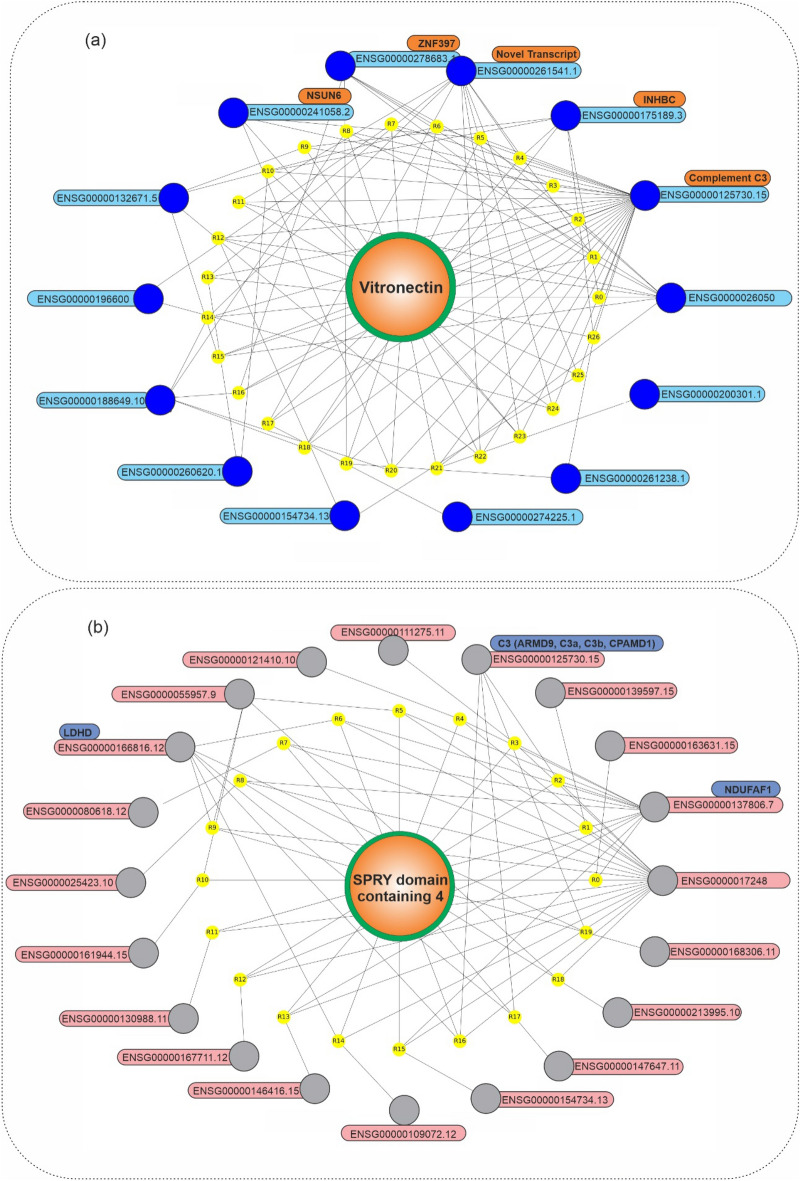
Graph network of Vitronectin and SPRY domain containing 4 in HCC. (**a**) Graph network of Vitronectin (with lift > 1.42) related association rules, in which the Vitronectin, its rules, and related mRNAs were presented, by orange, yellow, and blue colors, respectively. Vitronectin, the most frequent mRNA in the early-stage association rules, has a high dependency on ENSG00000125730 (Complement C3). (**b**) Graph network of the SPRY domain containing 4 (with lift > 1.46) related association rules, in which the SPRY domain containing 4, its rules, and related mRNAs were presented, by orange, yellow, and blue colors, respectively. Python programming language (version 3.9) and Matplotlib library (version 3.6.0) were used to draw the plots, all of them are open sources.

Vitronectin was the most frequent itemset in early-stage association rules (with 1533 repeat counts). Hence, to investigate its associations with other features, its relations based on association rules were studied in the graph network (Fig. 5). In Fig. 5a, it is noticeable that Vitronectin, the most frequent mRNA at the early-stage association rules, is dependent on ENSG00000125730 (Complement C3). Furthermore, the ENSG00000176422 (SPRY domain containing 4) was the most frequent itemset with 7297 repeat counts in late-stage association rules. In Fig. 5b, it is obvious that the SPRY domain containing 4, the most frequent mRNA in the late-stage association rules, is associated with ENSG00000017248, ENSG000000137806, and ENSG000000166816. More in-depth biological functions of these findings are provided in the “Discussion” section.

## Discussion and conclusion

Accurate prediction and stage classification of HCC are vital for the management of patients since the proper HCC treatment decisions are impacted by the degree of liver impairment and tumor stage. In this study, aberrantly expressed mRNA and microRNA patterns were identified by deep learning that can discriminate early stage from the late stage of cancerous HCC with high accuracy. Utilizing ARM analysis, top candidate mRNAs and microRNAs were found in early and late HCC association rules. Vitronectin, thrombin-activatable fibrinolysis inhibitor (TAFI), lactate dehydrogenase D (LDHD), and miR-590 were identified as top transcripts involved at the early stage of HCC. A SPRY domain containing 4, regucalcin, and miR-3199-1 were identified to play important roles at the late stage of HCC.

The crosstalk between cancer cells and their microenvironment is the first stage in the expansion of metastasis. In the present study, vitronectin was the first identified mRNA by association rule mining to be implicated at the early stage of HCC. Vitronectin is an adhesive multifunctional glycoprotein that links cells to the extracellular matrix (ECM) via different ligands such as urokinase plasminogen activator receptor (uPAR), plasminogen activator inhibitor-1 (PAI-1), and integrins. Vitronectin is mainly synthesized by hepatocytes^22^ and plays major roles in cell growth, cell adhesion, differentiation, progression, migration, regulation of the innate immune system, complement activation, and angiogenesis under different biological and pathological circumstances^23^. Moreover, vitronectin participates in other biological processes such as controlling tissue remodeling, wound healing, and coagulation pathway (fibrinolysis and thrombosis). Some tumor cells have been reported to secrete vitronectin^24,25^ to promote ECM degradation and cell migration.

The role of vitronectin in the pathogenesis of HCC has been reported previously. Cytokines and/or growth factors can stimulate the synthesis and secretion of vitronectin in hepatocarcinoma cells^26^ and promote the adhesion and migration of cancer cells^27^. Within the liver tumor microenvironment, expressed vitronectin can support the recruitment and preservation of effector lymphocytes by a uPAR-mechanism^24^. uPAR is an anchored receptor, interacting with uPA and some molecules, such as vitronectin and integrins. Evidence indicates that in different cancers, the uPAR-uPA system (by activating plasminogen and fibrinolysis) is linked with tumor progression, peritoneal dissemination, and metastasis^28^. Abnormal levels of uPAR might induce EMT by vitronectin binding and easing tumor invasion and metastasis^29^. An increased serum level of vitronectin represents high diagnostic and prognostic values for HCC^30^ since it is associated with clinicopathological factors and early recurrence^31^, cell migration^27^, and the malignant growth of the tumor. Vitronectin when freed from the cancer cells complex guarded by fibrinogen, functions as a pro-migratory factor for directing metastasis of cancer cells to low-fibrinogen body cavities or lymphatics in a uPAR-dependent manne^32^. Suppression of vitronectin can inhibit HCC in vitro and in vivo^33^, therefore, it can potentially be considered a therapeutic target for the treatment of HCC.

Cases with advanced HCC have irregular fibrinolysis and coagulation that is associated with tumor progression where cancer-associated thrombosis is an important cause of mortality. Venous thromboembolism, mainly portal vein tumor thrombus, is a challenging and common complication in the HCC that can be the earliest sign of an underlying malignancy^34,35^; it indicates a worse prognosis and less tolerance to treatment. In this regard, thrombin-activatable fibrinolysis inhibitor (TAFI) was identified as the second top transcript at an early stage of HCC by our analysis. TAFI, also called carboxypeptidase B2, is a plasma glycoprotein that is activated by plasmin or thrombin during the coagulation cascade. It acts as a molecular link between fibrinolysis and coagulation and can also regulate the interaction between inflammation and coagulation^36^. The binding of thrombin to thrombomodulin, a regulator of hemostasis that plays an anti-metastatic role in cancer, is essential for TAFI activation. An elevated level of TAFI is associated with several types of cancer and a more advanced cancer stage^37–39^, signifying that TAFI can play a role in the pathogenesis of thrombosis in cancer. Beyond activation of systemic coagulation, TAFI secretion from cancer cells elevates the intra-tumoral deposition of fibrin, promoting the growth and dissemination of tumor cells^40^. It is proposed that the production of TAFI can be mediated by directly malignant cells or indirectly by liver/endothelial cells that are induced by cancer-induced inflammatory cytokines. Modulation of TAFI may hinder migration and invasion of cancer cells^41,42^; therefore, TAFI can be another valuable molecular target for the treatment of HCC.

Lactate dehydrogenase D (LDHD) was the 3rd identified mRNA at the early stage of HCC in this study. It is responsible for the mitochondrial metabolism of D-lactate (a less common form of lactate) in humans^43^ and is supposed to produce by cancer cells^44^. The LDHD preferentially uses NADPH as a coenzyme that differs from the coenzyme that is used by other LDHs (A–C). In cancer cells, the mitochondrial LDHD metabolism is more active than in normal cells^45^ and its elevated level was detected in clear cell renal cell carcinoma^46^, prostate cancer^47^, and uterine sarcoma^48^. The methylglyoxal (MG) pathway produces an end-product, LDHD, to eliminate the toxic glycolysis-derived MG^49^, fatty acid synthesis, and scavenge reactive oxygen species, all of which are vital for cancer cell proliferation and viability^47^. Based on the available studies, D- lactate metabolism can represent a target for the development of an anticancer therapeutic strategy in the HCC.

NADH dehydrogenase 1 alpha subcomplex assembly factor 1 (*NDUFAF1*), the 4th identified mRNA, is a chaperone protein in mitochondria that are implicated in the assembly of the NADH^50^. Its downregulation is connected with the recurrence of HCC^51^.microRNAs (miRs) are non-coding, small RNAs that regulate gene expression negatively. Their abnormal expression, as oncogenes or tumor suppressors, is involved in the initiation, development, and metastasis of HCC. Evidence suggests that certain subsets of miRs can be therapeutic targets for HCC. In our association rule mining analysis, top miRs including miR-590, miR-23a, miR-4443, miR-3691, and miR-877 were identified to be involved at the early stage of the HCC, the roles of which have been reported previously. miR-590 plays a tumor suppressor role in HCC by targeting a variety of transcripts such as transcriptional enhancer activator domain 1 (TEAD1)^52^, Wnt pathway^53^, TGF-beta RII^54^, and ROCK2^55^. A bioinformatics analysis in HCC cell lines indicated that SOX2, CX3CL1, E-cadherin, N-cadherin, and FOXA2 are the potential downstream target genes of miR-590-3p in HCC^56^. This microRNA can be a potential target molecule for the treatment of HCC.

This work has some limitations. We did not validate the results on other cancer genomic datasets including, gene expression omnibus (GEO). It is suggested to validate the results in other datasets in future works. Further bioinformatics analysis is needed to be performed to find the targets of the identified microRNAs and to understand their correlations with the identified mRNAs.

Identification of therapeutic targets is essential for the effective development of drugs for HCC. In this study, we applied an AI-based framework to highlight putative mRNA and microRNA targets for HCC. The applied methods could identify key genes associated with the early (e.g., Vitronectin, TAFI, LDH-D, miR-590) and late-stage (e.g., SPRY domain containing 4, regucalcin, miR-3199-1, miR-194-2, miR-4999) of HCC. Applying targeted molecular therapy at an early stage and proper time will improve the outcome of patients with HCC and lessen their mortality rate. This research could establish a likely clear picture of putative candidate genes which could be the main actors at the early and late-stage of HCC.

## Methods

### Dataset

We obtained the mRNA and miRNA profiles of HCC samples from The Cancer Genome Atlas (TCGA) database; which is accessible in the GDC data portal (https://portal.gdc.cancer.gov/). Furthermore, clinical data were downloaded to extract the sample's HCC stage based on its Biospecimen Core Resource ID. The mRNA expression was reported in terms of FPKM values for 60,483 RNA transcripts. In the miRNA profile, 1881 miRNA expression values were recorded using the Illumina HiSeq 2000 platform. The HCC stage system was defined based on the TNM system; (T) the size of the primary tumor, (M) the distant metastasis, and (N) the spread of cancer to lymph nodes. In this study, we considered stage I as an early-stage class and stages II, III, and IV as a late-stage class. The information on mRNA/miRNA data is displayed in Table 5 in more detail.

**Table 5:** Information of mRNA and miRNA data.

mRNA data		miRNA data	
Early-stage	Late-stage	Early-stage	Late-stage
189	192	190	192

## Method

The whole process was displayed in Fig. 1. The proposed algorithms were applied to mRNA and miRNA data separately.

In the pre-processing step, first, we organized the mRNA and miRNA data into a matrix form with 381/382 rows and 60,483/1881 columns for mRNA/miRNA data that present the number of samples and features, respectively. Next, the nested cross-validation (CV) technique was applied to data for accurate error estimation in the real world. In the nested CV, the number of folds in the outer and inner loops were considered 10 and 5, respectively. Then, features with the same value in all samples of training folds of the inner loops were removed. Finally, z-score and min–max methods were applied for normalization in feature selection/classification and association rule mining steps, respectively. In the z-score, we mapped the distribution of features into the normal distribution, and in the min–max, we scaled features into the [0 1] range.

In the feature selection step, filter and wrapper methods were applied. The filter methods reduce the number of features (mRNAs/miRNAs) by removing the irrelevant attributes and decreasing computational cost and time for the wrapper step. These methods evaluate features individually in the selection procedure and are classifier-independent. In the filter method, T-test and ANOVA were used for mRNA and miRNA data based on their performance in feature selection, respectively. We applied filter methods to training folds of each inner loop, and we selected 25 top features based on their p-values. Next, this process was repeated ten (tenfold in outer-loop) products five (fivefold in inner-loop) times. Finally, 261 mRNAs and 150 miRNAs were selected based on the union of selected features obtained from training folds of inner-outer loops.

The wrapper methods considered the interaction of features and due to using the classifier in the selection procedure, they are classifier-dependent. We applied binary particles swarm optimization (PSO) for feature selection as a wrapper method. In binary PSO, the support vector machine (SVM) was utilized for the fitness function evaluation. Due to being robust and reliable, we defined the fitness function based on AUC, shown in Eq. (1). Besides, the fitness function value, including mean and standard deviation of AUC, was calculated based on inner validation folds of each outer fold. Ultimately, 123 significant mRNAs and 77 miRNAs were selected based on binary PSO output.

1 $$fitness\;value = \left( {1 - mean(AUC\; in\; all\; validation\;folds} \right) + mean\left( {standard\;deviation\;of\;AUC\;in\;all\;validation\;folds} \right)$$

In the classification step, we evaluated the discrimination power of selected features (mRNAs/miRNAs) by classifying early-late stages groups. The performance of the classifier represents how much-selected features are significant. SVM, random forest (RF), K-nearest neighbor (KNN), Naive Bayes (NB), Deep self-organizing auto-encoder (SOAE), logistic regression (LR), and XgBoost were used for the classification task. Furthermore, the performance of classifiers was reported using accuracy, AUC, MCC, F_1_-score, sensitivity, and specificity.

In the next step, significant relationships were discovered by association rule mining. We extracted association rules concerning selected mRNAs/miRNAs and early/late HCC stage groups. In this regard, the early/late stages group was added as a new feature to mRNA/miRNA data, and features (mRNA/miRNA) were categorized into three parts, namely low, medium, and high expression levels. Next, the FP-Growth algorithm was utilized to generate association rules in two phases, including frequent itemsets and rules generation. Next, early and late stages association rules were obtained based on the consequent part of rules with early and late-stage values, respectively. Then, we studied the antecedent part of early-stage association rules and reported mRNAs/miRNAs based on their repeat count in early-stage association rules. Finally, we studied five top miRNAs/mRNAs of early-stage and late-stage, based on the repeat count, more in-depth from a biological point of view.

The concept of the above algorithms was explained comprehensively in the following sections. In addition, the output of each step was reported and displayed in the “Result” section.

### Nested cross-validation

Feature selection and classification are the main actors in the machine learning and data mining areas. The quality of the classifier is dependent on the quality of selected features. The classifier performance is calculated based on testing data, which is not used for training and validating the model. Combining too many irrelevant features may lead to low generalization in testing data and high variance error estimation (overfitting) in the training process. In contrast, a lack of significant features may lead to high bias error estimation (underfitting). In this regard, the precise error estimation method plays a crucial role in classification and feature selection procedures.

Cross-validation (CV) is a fundamental action for the classifier accuracy/error estimation in a given dataset by splitting data into training and testing sets. Various versions of CV have been implemented to apply in feature selection and classifier parameters tuning, including leave-one-out CV, repeated double CV, and nested CV. The nested CV is a reliable way for classifier accuracy/error estimation^57^. The data is split into k outer folds in nCV and the remaining k-1 folds were merged and split into inner folds for inner training and validation. Training outer folds, including inner training folds and validation folds, are used for feature selection and model parameter tuning. Finally, general classifier accuracy/error is estimated based on testing outer folds.

### z-score and min–max normalization

Z-score and min–max normalization are implemented by Eqs. (2) and (3), respectively. In Eq. (2), µ and σ are the mean and standard deviation values of $x$, and in Eq. (3), min and max are the minima and maximum values of $x$ (feature). The Z-score process alters data distribution and converts it to normal. While the min–max method does not change data distribution and only scales data to the [0 1] range.

2 $$y= \frac{x-\mu }{\sigma }$$

3 $$y= \frac{1}{Max-Min}(x-Min)$$

### T-test and ANOVA

The t-test and ANOVA are a type of statistical tests employed to compare the mean of two groups. They are parametric statistical hypotheses, which are widely used in medical data. In parametric methods, there are some assumptions about the distribution of probability variables and parameters of the distribution. Conditions of normality, equal variance, and independence of samples are the principal assumptions in the t-test.

In the t-test method^58^, the t-statistic, based on Eq. (4), is calculated.

4 $$t= \frac{\left({\overline{x} }_{1}-{\overline{x} }_{2}\right)-({\mu }_{1}-{\mu }_{2})}{\sqrt{\frac{{S}_{1}^{2}}{{n}_{1}}+\frac{{S}_{2}^{2}}{{n}_{2}}}}$$

In Eq. (4), ${\overline{x} }_{i}$, ${S}_{i}^{2}$, ${n}_{i}$, and ${\mu }_{i}$ is the sample mean, sample variance, the number of samples, and ith population mean, respectively. Equation (4) converts to Eq. (5) by considering ${\mu }_{1}-{\mu }_{2}=0$ based on the null hypothesis.

5 $$t= \frac{\left({\overline{x} }_{1}-{\overline{x} }_{2}\right)}{\sqrt{\frac{{S}_{1}^{2}}{{n}_{1}}+\frac{{S}_{2}^{2}}{{n}_{2}}}}$$

In the ANOVA method^59^, F-statistic, based on the following steps, is calculated and compared with a threshold to find important features.

*Step 1* Calculating the variation between groups (Eqs. 6 and 7):

6 $$Between\;sum\;of\;squares\,\left( {BSS} \right) = \mathop \sum \limits_{i = 1}^{C} n_{i} \left( {\overline{x}_{i} - \overline{x}} \right)^{2}$$

7 $$Between\;mean\;squares\,\left( {BMS} \right) = \frac{BSS}{{df_{B} }}$$

where ${n}_{i}$, ${\overline{x} }_{i}$, and $\overline{x }$ are the number of samples and mean of samples in *i*th group, and mean of all samples, respectively. Also, ${df}_{B}=K-1$ is the degree of freedom.

*Step 2* Calculating the variation within groups (Eqs. 8 and 9):

8 $$Within\;sum\;of\;squares\,\left( {WSS} \right) = \mathop \sum \limits_{i = 1}^{C} \left( {n_{i} - 1} \right)\sigma_{i}^{2}$$

9 $$Within\;mean\;squares\,\left( {WMS} \right) = \frac{WSS}{{df_{w} }}$$

$\sigma$ is the standard deviation, and ${df}_{w} = (N - K)$ where $N$ and $K$ a are the number of total samples and groups, respectively.

*Step 3* Calculating F-test statistic (Eq. 10):

10 $$F= \frac{BMS}{WMS}$$

The F-statistic demonstrates features of discriminative capabilities and its higher values mean that the variation among means of groups is less likely to happen by chance.

### Particle swarm optimization (PSO)

Many algorithms utilize swarm intelligence to solve optimization problems, such as PSO, ant colony optimization (ACO), etc. PSO is a widely used algorithm for optimization among swarm intelligence-based algorithms^60^. It is well-known as an easy and flexible method from the implementation point of view. This algorithm uses a mathematical simulated model based on swarm behavior such as bird flocking in nature.

The PSO discovers the objective function space for finding optimum points by updating the position and tuning the velocity of individual agents, called particles. Updating of the particle position is implemented by Eq. (11). Adjusting of movement is defined by Eq. (12), composed of three components based on its own best location (${X}_{i}^{*}$), global best location (${g}^{*}$), and previous velocity (${v}_{i}^{t}$). In Eqs. (11) and (12), ${X}_{i}^{t}$ and ${v}_{i}^{t}$ are the position and velocity of *i*th particle in $t$ time, respectively.

11 $$X_{i}^{t + 1} = X_{i}^{t} + v_{i}^{t + 1}$$

12 $$v_{i}^{t + 1} = \theta v_{i}^{t} + \alpha \epsilon_{1} \left[ {g^{*} - X_{i}^{t} } \right] + \beta \epsilon_{2} \left[ {X_{i}^{*} - X_{i}^{t} } \right]$$

In Eq. (12), $\epsilon_{1}$ and $\epsilon_{2}$ are two random vectors, which the values of their elements are between 0 and 1. $\alpha$ and $\beta$ are user-defined parameters, which can typically be $\alpha \approx 2\;and\;\beta \approx 2$. $\theta$ is the value between 0 and 1, which in the simplest case is defined by $\theta \approx 0.5\sim 0.9$. The pseudo-code of PSO is illustrated in Table 6 in more detail.

**Table 6:**
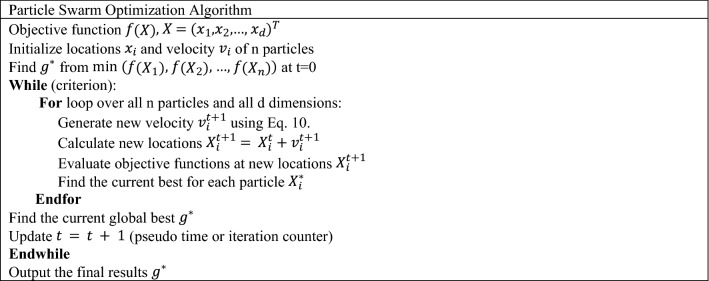
Pseudo code of particle swarm optimization^61^.

In the standard PSO, position and velocity are based on continuous values. However, many real-world optimization problems search space are defined based on discrete values, such as binary problems. In this regard, Kennedy and Eberhart presented a new version of standard PSO for discrete optimization in 1997^62^. They applied sigmoid and uniform transformations to the velocity vector and position vector, as shown in Eqs. (13) and (14).

13 $$S\left( {v_{i}^{k} } \right) = \frac{1}{{1 + {\text{exp}}\left( { - v_{i}^{k} } \right)}}\quad k = 1, 2, \ldots , d$$

14 $$x_{i}^{k} = \left\{ {\begin{array}{*{20}c} 1 & {if\;r < S\left( {v_{i}^{k} } \right)} \\ 0 & {otherwise} \\ \end{array} } \right.$$

where $r$ is a random variable in the [0 1] range. The value of each velocity element ${v}_{i}^{k}$ is defined as the probability of taking one value by ${x}_{i}^{k}$. The binary PSO (BPSO) significantly varies from the standard continuous PSO.

### Classification

Classifier models are powerful tools that apply well-known machine-learning algorithms for classification tasks. In this study, we used SVM, NB, KNN, RF, LR, and XgBoost as classic classifiers, and Deep self-organizing auto-encoder (SOAE)^63^ to assess the discrimination power of selected features.

The predictive performance of the classifier was evaluated using the following evaluation metrics (Eqs. 15–19), including accuracy, F_1_-score, Matthews correlation coefficient (MCC), sensitivity (Sn), and specificity (Sp). False negative (FN), false positive (FP), true negative (TN), and true positive (TP).

15 $$Accuracy= \frac{TP+TN}{TP+TN+FP+FN}\times 100$$

16 $$MCC=\frac{TP\times TN-FP\times FN}{\sqrt{(TP+FP)(TP+FN)(TN+FP)(TN+FN)}}$$

17 $${F}_{1}=\frac{TP}{TP+\frac{1}{2}(FP+FN)}$$

18 $$sensitivity= \frac{TP}{TP+FN}\times 100$$

19 $$specificity=\frac{TN}{TN+FP}\times 100$$

### Association rule mining

Association rule mining is a potent data mining tool that presents the hidden association in the form of rules by discovering associated frequently co-occurring items in the dataset. Market basket analysis^64–66^ and bioinformatics^67^ are two main areas that apply association rule mining for the extraction of significant associations in marketing and genomic data, respectively. The interpretation of gene expression data (mRNA/miRNA), annotations, detection of protein interaction, and biomolecular localization prediction are some applications of association rule mining in bioinformatics^67^.

Association rule mining has two main steps, frequent itemset mining (FIM) and association rule generation. FIM extracts frequently co-occur sets of items (i.e., frequent itemsets). If itemset support is more than the minimum support threshold, itemset is called a frequent itemset. Next, the association rule generation step creates the rules from the discovered frequent itemsets (FIs). If the support/confidence/lift of the rule is no less than the minimum support/confidence/lift threshold, the generated rule is called the association rule. These thresholds are user-defined parameters.

Association rule mining is an NP-hard problem, in which finding the results is challenging in a reasonable time. Introducing the Apriori algorithm addressed the computational problem in most regular-sized data^68^. Since then, many types of research have been done to develop new algorithms such as FP-Growth^69^ and Eclat^70^. These algorithms improved the scalability of the Apriori algorithm. However, the computational cost of association rule mining in the FIM stage for high-dimension data and big data is a challenging subject.

There are some principal terms in association rule mining, which are mentioned in the above section. In the following, we describe and formalize these basic concepts of frequent itemset and association rules. The related theories are available in^71^ with more details. Let $I=\{{i}_{1}, {i}_{2}, \dots ,{i}_{d}, y\}$ is a set of items, $D=\{{d}_{1}, {d}_{2}, \dots , {d}_{n}\}$ is a dataset of n instances, $F=\{{f}_{1}, {f}_{2}, \dots , {f}_{m}\}$ is the features space with m features, and $Y=\{0, 1\}$ is the user-defined phenotype. The ${d}_{i}$ can be presented as a tuple $({X}_{i}, {y}_{i})$, where ${X}_{i}\in {f}_{1}\times {f}_{2}\times \dots \times {f}_{m}$ and ${y}_{i}\in Y$.

#### *Definition 1.*

(Length of an itemset)

Let $X$ be an itemset, which has K-distinct items, the length of the $X$ is defined as $|X|=K$.

#### *Definition 2.*

(Support count and support of an itemset)

The total number of samples including $X$ itemset is defined support count of an itemset $X$. Also, support of an item set $X$ is the ratio of support count to the total number of samples.

#### *Definition 3.*

(Frequent itemset)

An itemset $X$ is called a frequent itemset if and only if its support is no less than the minimum support, which is the user-defined threshold.

#### *Definition 4.*

(Association rule)

An association rule is defined as a form of $A\to C$, where $A$ and $C$ are itemsets and $A\cup C=\varphi$, $A\subset X, C\subset X$. In the $A\to C$, $A$ and $C$ are called the Antecedent and Consequent, respectively. Also, $A\to C$ displays the association that if all items in Antecedent occur, then all items in Consequent co-occur. The generated association rules are filtered out based on the user-defined threshold, such as support, confidence, and lift.

#### *Definition 5.*

(Support of rule)

The support of rule $A\to C$ is the percentage of samples in D (as shown in Eq. 20). This measure presents the usefulness of the rule.

20 $$Support\left(A\to C\right)= \frac{support(A\cup C)}{n}$$

#### *Definition 6.*

(Confidence of rule)

The confidence of rule $A\to C$ is the percentage value that displays how frequently $C$ occurs among all the examples containing $A$ (as shown in Eq. 21). This measure shows the certitude of the rule.

21 $$Confidence\left(A\to C\right)=P\left(C|A\right)= \frac{support(A\cup C)}{support(A)}$$

#### *Definition 7.*

(Lift of rule)

The Lift of rule $A\to C$ defines that the occurrence of itemsets $A$ is dependent to the $C$. When the Lift value is less (more) than 1, the occurrence of $A$ is negatively (positively) associated with the occurrence of $C$. $A$ and $C$ are independent when the Lift value is equal to 1. The Lift value is shown in Eq. (22).

22 $$Lift\left(A\to C\right)= \frac{P(A\cup C)}{P\left(A\right)P(C)}$$

Here the FP-Growth approach was applied for association rule mining. Also, the pseudo-codes of the two stages are available in Tables 7 and 8.

**Table 7:**
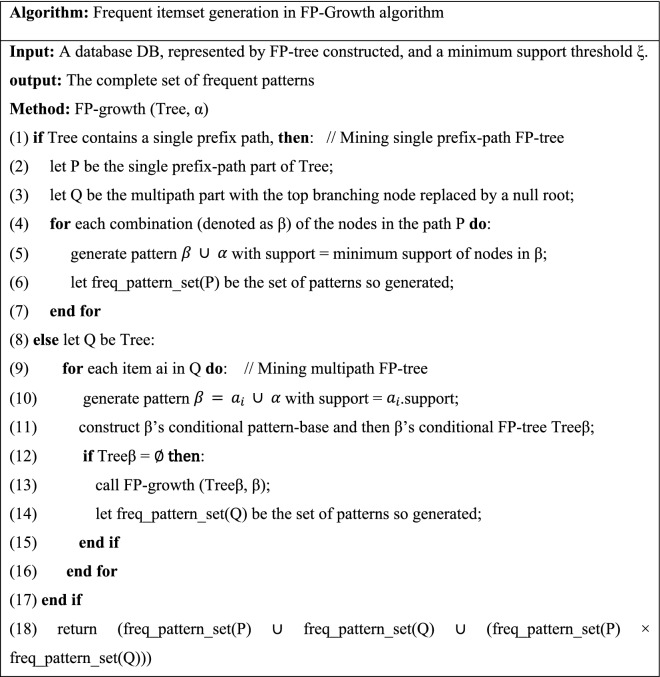
Pseudocode of frequent itemset generation step in FP-Growth algorithm^69^.

**Table 8:**
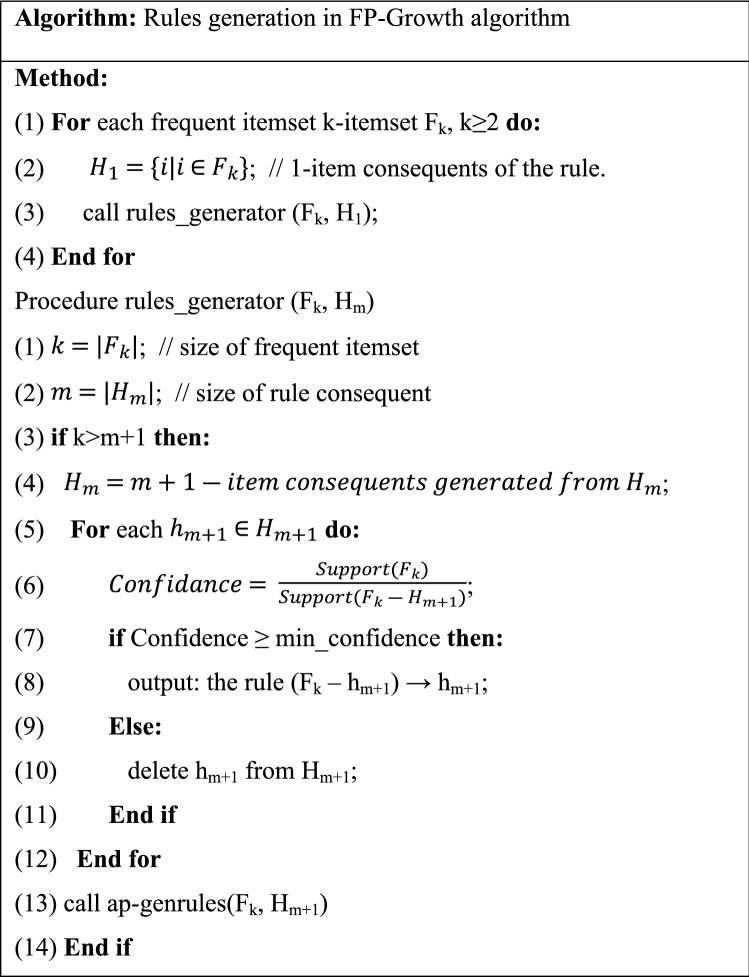
Pseudocode of rules generation step in FP-Growth algorithm^72^.

### Ethical approval

This study was approved by the Ethics Committee of Tabriz University of Medical Sciences, Tabriz, Iran (Ethical code: IR.TBZMED.VCR.REC.1400.291).

## Supplementary Information

Supplementary Information.

## Data Availability

The data obtained from the artificial intelligence approaches will be available from the corresponding authors upon request.
