# Supplementary material for: Key therapeutic targets implicated at the early stage of hepatocellular carcinoma identified through machine-learning approaches
Source: Sci Rep. 2023 Mar 7;13:3840. doi: 10.1038/s41598-023-30720-x (PMC9992672; doi:10.1038/s41598-023-30720-x)
Supplement: Supplementary file 1 — Supplementary Information. [file 41598_2023_30720_MOESM1_ESM.docx]

**Key therapeutic targets implicated at the early stage of hepatocellular carcinoma identified through machine-learning approaches**

Seyed Mahdi Hosseiniyan Khatibi, PhD^1, 2, 4 †^, Farima Najjarian, MD^3 †^, Hamed Homaei Rad, Msc^4^, Mohammadreza Ardalan, MD^1^, Mohammad Teshnehlab, PhD^5^, Sepideh Zununi Vahed, PhD ^1^ *, Saeed Pirmoradi, PhD ^2^ *

^1^ Kidney Research Center, Tabriz University of Medical Sciences, Tabriz, Iran

^2^ Clinical Research Development Unit of Tabriz Valiasr Hospital, Tabriz University of Medical Sciences, Tabriz, Iran

^3^ Faculty of Medicine, Tabriz University of Medical Sciences, Tabriz, Iran.

^4^ Rahat Breath and Sleep Research Center, Tabriz University of Medical Science, Tabriz, Iran.

^5^ Department of Electric and Computer Engineering, K.N. Toosi University of Technology, Tehran, Iran.

***Corresponding authors:**

Sepideh Zununi Vahed

Kidney Research Center, Tabriz University of Medical Sciences, Tabriz, Iran.

Daneshgah Street, Postal code 51665118, Tabriz, Iran.

Email: [sepide.zununi@gmail.com](mailto:sepide.zununi@gmail.com)

Saeed Pirmoradi

Clinical Research Development Unit of Tabriz Valiasr Hospital, Tabriz University of Medical Sciences, Tabriz, Iran.

Niyayesh Blvd., Tabriz, Iran.

Email: [said.pirmoradi@gmail.com](mailto:said.pirmoradi@gmail.com)

† These authors contributed equally.

**Supplementary Tables**

**Table S1.** List of selected miRNAs in feature selection step

| No. | miRNA ID | No. | miRNA ID | No. | miRNA ID |
| --- | --- | --- | --- | --- | --- |
| 1 | hsa-let-7e | 27 | hsa-mir-330 | 53 | hsa-mir-5090 |
| 2 | hsa-mir-124-1 | 28 | hsa-mir-331 | 54 | hsa-mir-545 |
| 3 | hsa-mir-1246 | 29 | hsa-mir-3622a | 55 | hsa-mir-548s |
| 4 | hsa-mir-1254-2 | 30 | hsa-mir-3680-1 | 56 | hsa-mir-548v |
| 5 | hsa-mir-1255a | 31 | hsa-mir-3691 | 57 | hsa-mir-561 |
| 6 | hsa-mir-1257 | 32 | hsa-mir-3926-1 | 58 | hsa-mir-5706 |
| 7 | hsa-mir-126 | 33 | hsa-mir-3926-2 | 59 | hsa-mir-576 |
| 8 | hsa-mir-1266 | 34 | hsa-mir-3934 | 60 | hsa-mir-590 |
| 9 | hsa-mir-1289-1 | 35 | hsa-mir-3936 | 61 | hsa-mir-629 |
| 10 | hsa-mir-1292 | 36 | hsa-mir-423 | 62 | hsa-mir-641 |
| 11 | hsa-mir-141 | 37 | hsa-mir-4435-1 | 63 | hsa-mir-643 |
| 12 | hsa-mir-147b | 38 | hsa-mir-4443 | 64 | hsa-mir-6515 |
| 13 | hsa-mir-149 | 39 | hsa-mir-4478 | 65 | hsa-mir-658 |
| 14 | hsa-mir-151b | 40 | hsa-mir-4487 | 66 | hsa-mir-6728 |
| 15 | hsa-mir-15b | 41 | hsa-mir-4523 | 67 | hsa-mir-6783 |
| 16 | hsa-mir-183 | 42 | hsa-mir-4526 | 68 | hsa-mir-6801 |
| 17 | hsa-mir-190b | 43 | hsa-mir-4654 | 69 | hsa-mir-6845 |
| 18 | hsa-mir-1911 | 44 | hsa-mir-4673 | 70 | hsa-mir-6888 |
| 19 | hsa-mir-194-2 | 45 | hsa-mir-4735 | 71 | hsa-mir-7-2 |
| 20 | hsa-mir-21 | 46 | hsa-mir-4746 | 72 | hsa-mir-765 |
| 21 | hsa-mir-216b | 47 | hsa-mir-4752 | 73 | hsa-mir-7850 |
| 22 | hsa-mir-22 | 48 | hsa-mir-4757 | 74 | hsa-mir-877 |
| 23 | hsa-mir-23a | 49 | hsa-mir-4764 | 75 | hsa-mir-885 |
| 24 | hsa-mir-3155a | 50 | hsa-mir-4771-2 | 76 | hsa-mir-940 |
| 25 | hsa-mir-3176 | 51 | hsa-mir-4798 | 77 | hsa-mir-95 |
| 26 | hsa-mir-3199-1 | 52 | hsa-mir-4999 |  |  |

**Table S2.** List of selected mRNAs in feature selection step

| **No.** | **mRNA ID** | **No.** | **mRNA ID** | **No.** | **mRNA ID** |
| --- | --- | --- | --- | --- | --- |
| 1 | ENSG00000010319.5 | 43 | ENSG00000161944.15 | 85 | ENSG00000228141.5 |
| 2 | ENSG00000025423.10 | 44 | ENSG00000162460.6 | 86 | ENSG00000229740.1 |
| 3 | ENSG00000036473.6 | 45 | ENSG00000162882.13 | 87 | ENSG00000230490.2 |
| 4 | ENSG00000055957.9 | 46 | ENSG00000163631.15 | 88 | ENSG00000230647.1 |
| 5 | ENSG00000072163.17 | 47 | ENSG00000163815.5 | 89 | ENSG00000231473.2 |
| 6 | ENSG00000073146.14 | 48 | ENSG00000166265.10 | 90 | ENSG00000231690.2 |
| 7 | ENSG00000080618.12 | 49 | ENSG00000166816.12 | 91 | ENSG00000233387.1 |
| 8 | ENSG00000084110.9 | 50 | ENSG00000167701.12 | 92 | ENSG00000236213.1 |
| 9 | ENSG00000091583.9 | 51 | ENSG00000167711.12 | 93 | ENSG00000237702.2 |
| 10 | ENSG00000109072.12 | 52 | ENSG00000168234.11 | 94 | ENSG00000241058.2 |
| 11 | ENSG00000111275.11 | 53 | ENSG00000168306.11 | 95 | ENSG00000241307.1 |
| 12 | ENSG00000113263.11 | 54 | ENSG00000170989.8 | 96 | ENSG00000242220.5 |
| 13 | ENSG00000114054.12 | 55 | ENSG00000172482.4 | 97 | ENSG00000243896.3 |
| 14 | ENSG00000118137.8 | 56 | ENSG00000173269.12 | 98 | ENSG00000244414.5 |
| 15 | ENSG00000120278.13 | 57 | ENSG00000174059.15 | 99 | ENSG00000245164.5 |
| 16 | ENSG00000120279.6 | 58 | ENSG00000174990.4 | 100 | ENSG00000245954.5 |
| 17 | ENSG00000121410.10 | 59 | ENSG00000175189.3 | 101 | ENSG00000246084.2 |
| 18 | ENSG00000124203.5 | 60 | ENSG00000175600.14 | 102 | ENSG00000248752.1 |
| 19 | ENSG00000125246.14 | 61 | ENSG00000176422.12 | 103 | ENSG00000255776.1 |
| 20 | ENSG00000125430.7 | 62 | ENSG00000176974.16 | 104 | ENSG00000255987.1 |
| 21 | ENSG00000125730.15 | 63 | ENSG00000178301.3 | 105 | ENSG00000258001.1 |
| 22 | ENSG00000128311.12 | 64 | ENSG00000178343.4 | 106 | ENSG00000259124.1 |
| 23 | ENSG00000130201.6 | 65 | ENSG00000180383.3 | 107 | ENSG00000260507.1 |
| 24 | ENSG00000130303.11 | 66 | ENSG00000182902.12 | 108 | ENSG00000260620.1 |
| 25 | ENSG00000130307.10 | 67 | ENSG00000185305.9 | 109 | ENSG00000261238.1 |
| 26 | ENSG00000130988.11 | 68 | ENSG00000188338.13 | 110 | ENSG00000261541.1 |
| 27 | ENSG00000132671.5 | 69 | ENSG00000188649.10 | 111 | ENSG00000261544.1 |
| 28 | ENSG00000134240.10 | 70 | ENSG00000196600.11 | 112 | ENSG00000262950.1 |
| 29 | ENSG00000137806.7 | 71 | ENSG00000197245.4 | 113 | ENSG00000264419.1 |
| 30 | ENSG00000139597.15 | 72 | ENSG00000197921.5 | 114 | ENSG00000264468.1 |
| 31 | ENSG00000141505.10 | 73 | ENSG00000200301.1 | 115 | ENSG00000270412.1 |
| 32 | ENSG00000145192.11 | 74 | ENSG00000204710.2 | 116 | ENSG00000270990.1 |
| 33 | ENSG00000146416.15 | 75 | ENSG00000211482.1 | 117 | ENSG00000272789.1 |
| 34 | ENSG00000146521.8 | 76 | ENSG00000211713.3 | 118 | ENSG00000273328.4 |
| 35 | ENSG00000147647.11 | 77 | ENSG00000211749.1 | 119 | ENSG00000274225.1 |
| 36 | ENSG00000151655.16 | 78 | ENSG00000211751.6 | 120 | ENSG00000275152.3 |
| 37 | ENSG00000154734.13 | 79 | ENSG00000212293.1 | 121 | ENSG00000278683.1 |
| 38 | ENSG00000157103.9 | 80 | ENSG00000213727.3 | 122 | ENSG00000279841.1 |
| 39 | ENSG00000157379.12 | 81 | ENSG00000213995.10 | 123 | ENSG00000279954.1 |
| 40 | ENSG00000158874.10 | 82 | ENSG00000225968.6 |  |  |
| 41 | ENSG00000160282.12 | 83 | ENSG00000226442.2 |  |  |
| 42 | ENSG00000161031.11 | 84 | ENSG00000226580.1 |  |  |

**Table S3. Twenty of top miRNAs early-stage rules based on sorted lift value**

| No. | Antecedent | | | | | | | Consequent |
| --- | --- | --- | --- | --- | --- | --- | --- | --- |
| 1 | if | has-mir-6515 | & | has-mir-590 | & | has-mir-23a | then | Early Stage |
| 2 | if | has-mir-6515 | & | has-mir-590 | & | has-mir-4443 | then | Early Stage |
| 3 | if | has-mir-4443 | & | has-mir-590 | & | has-mir-23a | then | Early Stage |
| 4 | if | has-mir-4764 | & | has-mir-590 | & | has-mir-23a | then | Early Stage |
| 5 | if | has-mir-7850 | & | has-mir-590 | & | has-mir-23a | then | Early Stage |
| 6 | if | has-mir-590 | & | has-mir-1289-1 | & | has-mir-23a | then | Early Stage |
| 7 | if | has-mir-590 | & | has-mir-6801 | & | has-mir-23a | then | Early Stage |
| 8 | if | has-mir-23a | & | has-mir-590 | & | has-mir-4487 | then | Early Stage |
| 9 | if | has-mir-590 | & | has-mir-4752 | & | has-mir-23a | then | Early Stage |
| 10 | if | has-mir-6515 | & | has-let-7e | & | has-mir-590 | then | Early Stage |
| 11 | if | has-mir-590 | & | has-mir-1255a | & | has-mir-23a | then | Early Stage |
| 12 | if | has-mir-590 | & | has-mir-4478 | & | has-mir-23a | then | Early Stage |
| 13 | if | has-mir-590 | & | has-mir-877 | & | has-mir-23a | then | Early Stage |
| 14 | if | has-mir-5090 | & | has-mir-590 | & | has-mir-23a | then | Early Stage |
| 15 | if | has-mir-590 | & | has-mir-331 | & | has-mir-4443 | then | Early Stage |
| 16 | if | has-mir-590 | & | has-mir-95 | & | has-mir-23a | then | Early Stage |
| 17 | if | has-mir-6515 | & | has-mir-590 | & | has-mir-1289-1 | then | Early Stage |
| 18 | if | has-mir-183 | & | has-mir-590 | & | has-mir-23a | then | Early Stage |
| 19 | if | has-mir-423 | & | has-mir-590 | & | has-mir-23a | then | Early Stage |
| 20 | if | has-mir-4764 | & | has-mir-590 | & | has-mir-4443 | then | Early Stage |

**Table S4. Twenty of top miRNAs late-stage rules based on sorted lift value**

| No. | Antecedent | | | | | | | Consequent |
| --- | --- | --- | --- | --- | --- | --- | --- | --- |
| 1 | if | hsa-mir-3199-1 | & | hsa-mir-194-2 | & | hsa-mir-885 | then | Late Stage |
| 2 | if | hsa-mir-3199-1 | & | hsa-mir-4654 | & | hsa-mir-194-2 | then | Late Stage |
| 3 | if | hsa-mir-3199-1 | & | hsa-mir-4999 | & | hsa-mir-194-2 | then | Late Stage |
| 4 | if | hsa-mir-3199-1 | & | hsa-mir-194-2 | & | hsa-mir-216b | then | Late Stage |
| 5 | if | hsa-mir-3199-1 | & | hsa-mir-126 | & | hsa-mir-194-2 | then | Late Stage |
| 6 | if | hsa-mir-3199-1 | & | hsa-mir-151b | & | hsa-mir-194-2 | then | Late Stage |
| 7 | if | hsa-mir-3199-1 | & | hsa-mir-3926-1 | & | hsa-mir-194-2 | then | Late Stage |
| 8 | if | hsa-mir-3199-1 | & | hsa-mir-3622a | & | hsa-mir-194-2 | then | Late Stage |
| 9 | if | hsa-mir-3199-1 | & | hsa-mir-6728 | & | hsa-mir-194-2 | then | Late Stage |
| 10 | if | hsa-mir-3199-1 | & | hsa-mir-194-2 | & | hsa-mir-641 | then | Late Stage |
| 11 | if | hsa-mir-3199-1 | & | hsa-mir-194-2 | & | ----------------- | then | Late Stage |
| 12 | if | hsa-mir-3199-1 | & | hsa-mir-330 | & | hsa-mir-194-2 | then | Late Stage |
| 13 | if | hsa-mir-3199-1 | & | hsa-mir-194-2 | & | hsa-mir-545 | then | Late Stage |
| 14 | if | hsa-mir-3199-1 | & | hsa-mir-4526 | & | hsa-mir-194-2 | then | Late Stage |
| 15 | if | hsa-mir-3199-1 | & | hsa-mir-548v | & | hsa-mir-194-2 | then | Late Stage |
| 16 | if | hsa-mir-3199-1 | & | hsa-mir-194-2 | & | hsa-mir-190b | then | Late Stage |
| 17 | if | hsa-mir-3199-1 | & | hsa-mir-194-2 | & | hsa-mir-3936 | then | Late Stage |
| 18 | if | hsa-mir-3199-1 | & | hsa-mir-194-2 | & | hsa-mir-3926-2 | then | Late Stage |
| 19 | if | hsa-mir-3199-1 | & | hsa-mir-3155a | & | hsa-mir-194-2 | then | Late Stage |
| 20 | if | hsa-mir-3199-1 | & | hsa-mir-4673 | & | hsa-mir-194-2 | then | Late Stage |

**Table S5. Twenty of top mRNAs early-stage rules based on sorted lift value**

| No. | Antecedent | | | | | | | Consequent |
| --- | --- | --- | --- | --- | --- | --- | --- | --- |
| 1 | if | ENSG00000109072.12 | & | ENSG00000130307.10 | & | ENSG00000175600.14 | then | Early Stage |
| 2 | if | ENSG00000109072.12 | & | ENSG00000113263.11 | & | ENSG00000175600.14 | then | Early Stage |
| 3 | if | ENSG00000109072.12 | & | ENSG00000204710.2 | & | ENSG00000175600.14 | then | Early Stage |
| 4 | if | ENSG00000124203.5 | & | ENSG00000109072.12 | & | ENSG00000175600.14 | then | Early Stage |
| 5 | if | ENSG00000109072.12 | & | ENSG00000175600.14 | & | --------------------------- | then | Early Stage |
| 6 | if | ENSG00000163815.5 | & | ENSG00000109072.12 | & | ENSG00000175600.14 | then | Early Stage |
| 7 | if | ENSG00000109072.12 | & | ENSG00000255987.1 | & | ENSG00000175600.14 | then | Early Stage |
| 8 | if | ENSG00000109072.12 | & | ENSG00000114054.12 | & | ENSG00000160282.12 | then | Early Stage |
| 9 | if | ENSG00000236213.1 | & | ENSG00000109072.12 | & | ENSG00000175600.14 | then | Early Stage |
| 10 | if | ENSG00000109072.12 | & | ENSG00000211751.6 | & | ENSG00000175600.14 | then | Early Stage |
| 11 | if | ENSG00000175600.14 | & | ENSG00000109072.12 | & | ENSG00000211749.1 | then | Early Stage |
| 12 | if | ENSG00000109072.12 | & | ENSG00000246084.2 | & | ENSG00000175600.14 | then | Early Stage |
| 13 | if | ENSG00000175600.14 | & | ENSG00000109072.12 | & | ENSG00000245954.5 | then | Early Stage |
| 14 | if | ENSG00000109072.12 | & | ENSG00000245164.5 | & | ENSG00000175600.14 | then | Early Stage |
| 15 | if | ENSG00000233387.1 | & | ENSG00000109072.12 | & | ENSG00000175600.14 | then | Early Stage |
| 16 | if | ENSG00000237702.2 | & | ENSG00000109072.12 | & | ENSG00000175600.14 | then | Early Stage |
| 17 | if | ENSG00000130303.11 | & | ENSG00000109072.12 | & | ENSG00000160282.12 | then | Early Stage |
| 18 | if | ENSG00000197245.4 | & | ENSG00000109072.12 | & | ENSG00000010319.5 | then | Early Stage |
| 19 | if | ENSG00000109072.12 | & | ENSG00000130307.10 | & | ENSG00000160282.12 | then | Early Stage |
| 20 | if | ENSG00000130201.6 | & | ENSG00000109072.12 | & | ENSG00000010319.5 | then | Early Stage |

**Table S6. Twenty of top mRNAs late-stage rules based on sorted lift value**

| No. | Antecedent | | | | | | | Consequent |
| --- | --- | --- | --- | --- | --- | --- | --- | --- |
| 1 | if | ENSG00000166816.12 | & | ENSG00000170989.8 | & | ENSG00000055957.9 | then | Late Stage |
| 2 | if | ENSG00000080618.12 | & | ENSG00000055957.9 | & | ENSG00000173269.12 | then | Late Stage |
| 3 | if | ENSG00000172482.4 | & | ENSG00000055957.9 | & | ENSG00000178301.3 | then | Late Stage |
| 4 | if | ENSG00000080618.12 | & | ENSG00000055957.9 | & | ENSG00000170989.8 | then | Late Stage |
| 5 | if | ENSG00000055957.9 | & | ENSG00000178301.3 | & | ENSG00000167701.12 | then | Late Stage |
| 6 | if | ENSG00000055957.9 | & | ENSG00000146416.15 | & | ENSG00000130988.11 | then | Late Stage |
| 7 | if | ENSG00000080618.12 | & | ENSG00000055957.9 | & | ENSG00000178301.3 | then | Late Stage |
| 8 | if | ENSG00000166816.12 | & | ENSG00000173269.12 | & | ENSG00000055957.9 | then | Late Stage |
| 9 | if | ENSG00000125730.15 | & | ENSG00000188338.13 | & | ENSG00000178301.3 | then | Late Stage |
| 10 | if | ENSG00000176422.12 | & | ENSG00000055957.9 | & | ENSG00000170989.8 | then | Late Stage |
| 11 | if | ENSG00000172482.4 | & | ENSG00000055957.9 | & | ENSG00000170989.8 | then | Late Stage |
| 12 | if | ENSG00000147647.11 | & | ENSG00000055957.9 | & | ENSG00000166816.12 | then | Late Stage |
| 13 | if | ENSG00000055957.9 | & | ENSG00000213995.10 | & | ENSG00000130988.11 | then | Late Stage |
| 14 | if | ENSG00000055957.9 | & | ENSG00000170989.8 | & | ENSG00000130988.11 | then | Late Stage |
| 15 | if | ENSG00000055957.9 | & | ENSG00000130988.11 | & | ENSG00000178301.3 | then | Late Stage |
| 16 | if | ENSG00000147647.11 | & | ENSG00000055957.9 | & | ENSG00000178301.3 | then | Late Stage |
| 17 | if | ENSG00000166816.12 | & | ENSG00000055957.9 | & | ENSG00000130988.11 | then | Late Stage |
| 18 | if | ENSG00000080618.12 | & | ENSG00000055957.9 | & | ENSG00000167701.12 | then | Late Stage |
| 19 | if | ENSG00000055957.9 | & | ENSG00000241307.1 | & | ENSG00000130988.11 | then | Late Stage |
| 20 | if | ENSG00000055957.9 | & | ENSG00000173269.12 | & | ENSG00000178301.3 | then | Late Stage |

**Supplementary Figures**


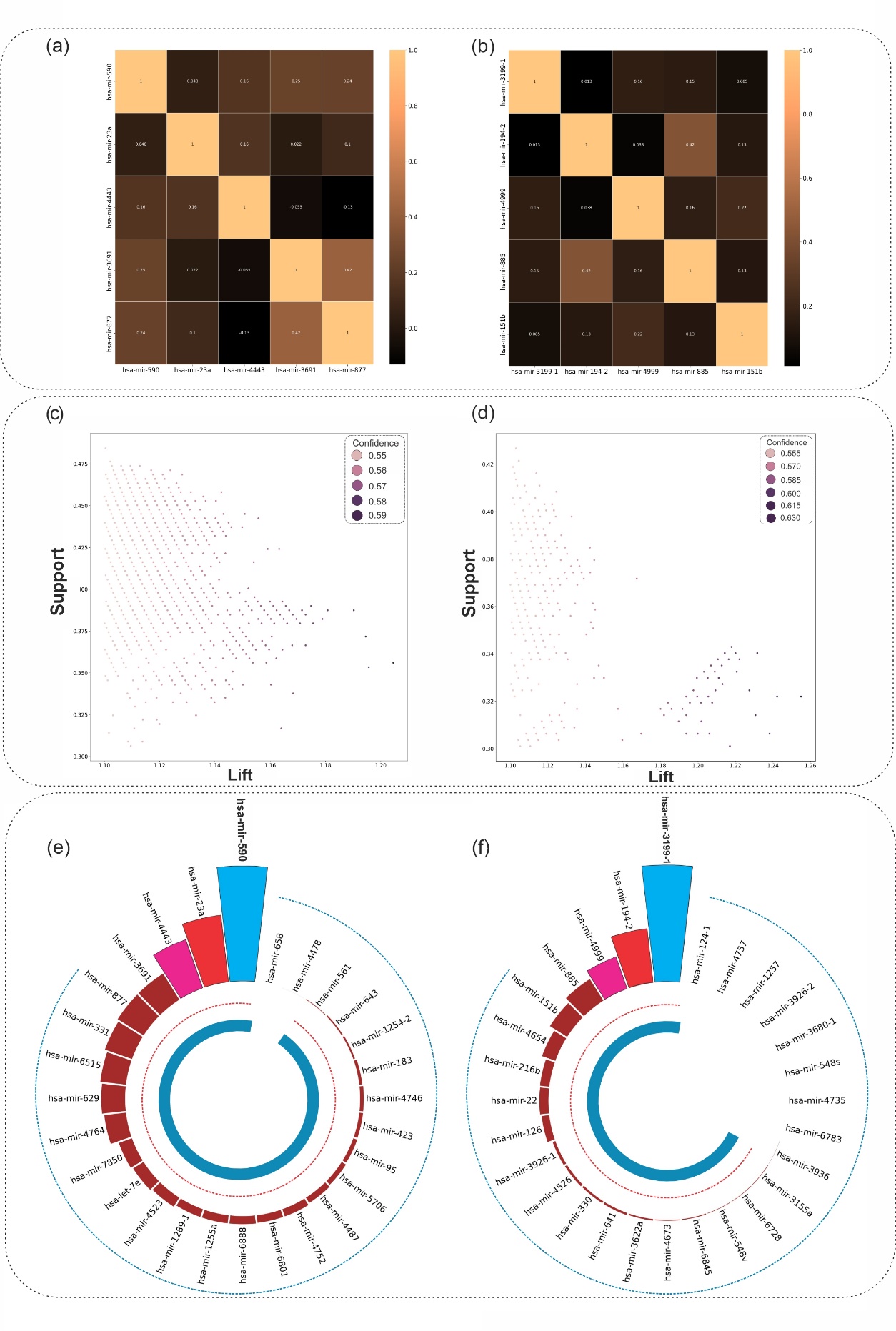


**Figure S1. Identified miRNAs at the early and late stages of HCC.** The heatmap plot based on the Spearman correlation for five top miRNAs of a) early-stage and b) late-stage rules. Strength distribution of c) early-stage and d) late-stage association rules according to their support, lift, and confidence. Ring bar plot of the repeat count of 28 top miRNAs in e) early-stage and f) late-stage rules. Python programming language (version 3.9) and Matplotlib library (version 3.6.0) were used to draw the heatmap plot, all of them are open sources.


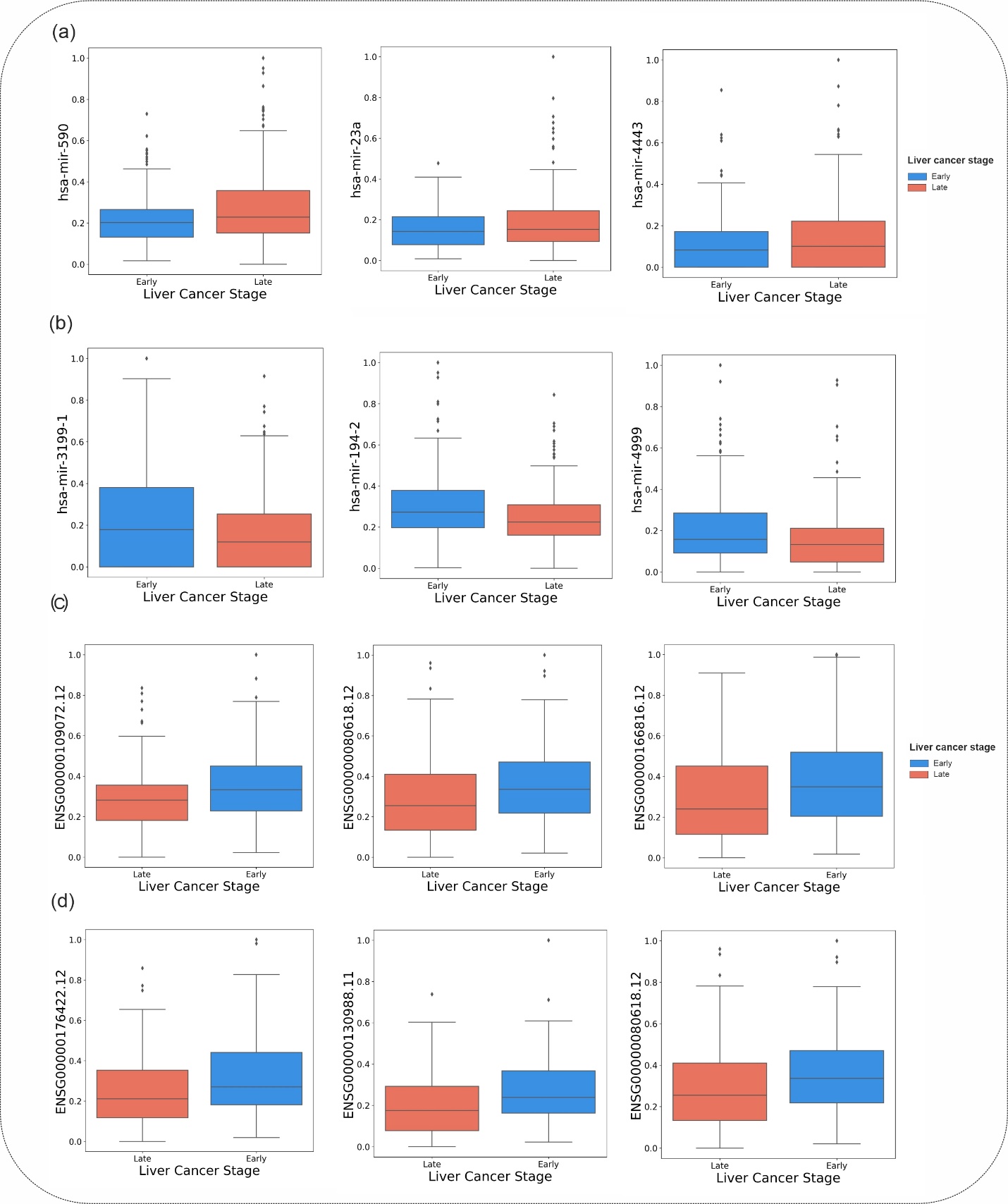


**Figure S2. The selected miRNA and mRNA are based on the association rule mining step.** Box plot of three top selected mRNAs based on a) early-stage association rules and b) late stage association rules. Box plot of three top selected miRNAs based on c) early stage association rules and d) late stage association rules. Expression values of miRNAs and mRNAs were normalized in the range of 0 to 1 by the min-max method.


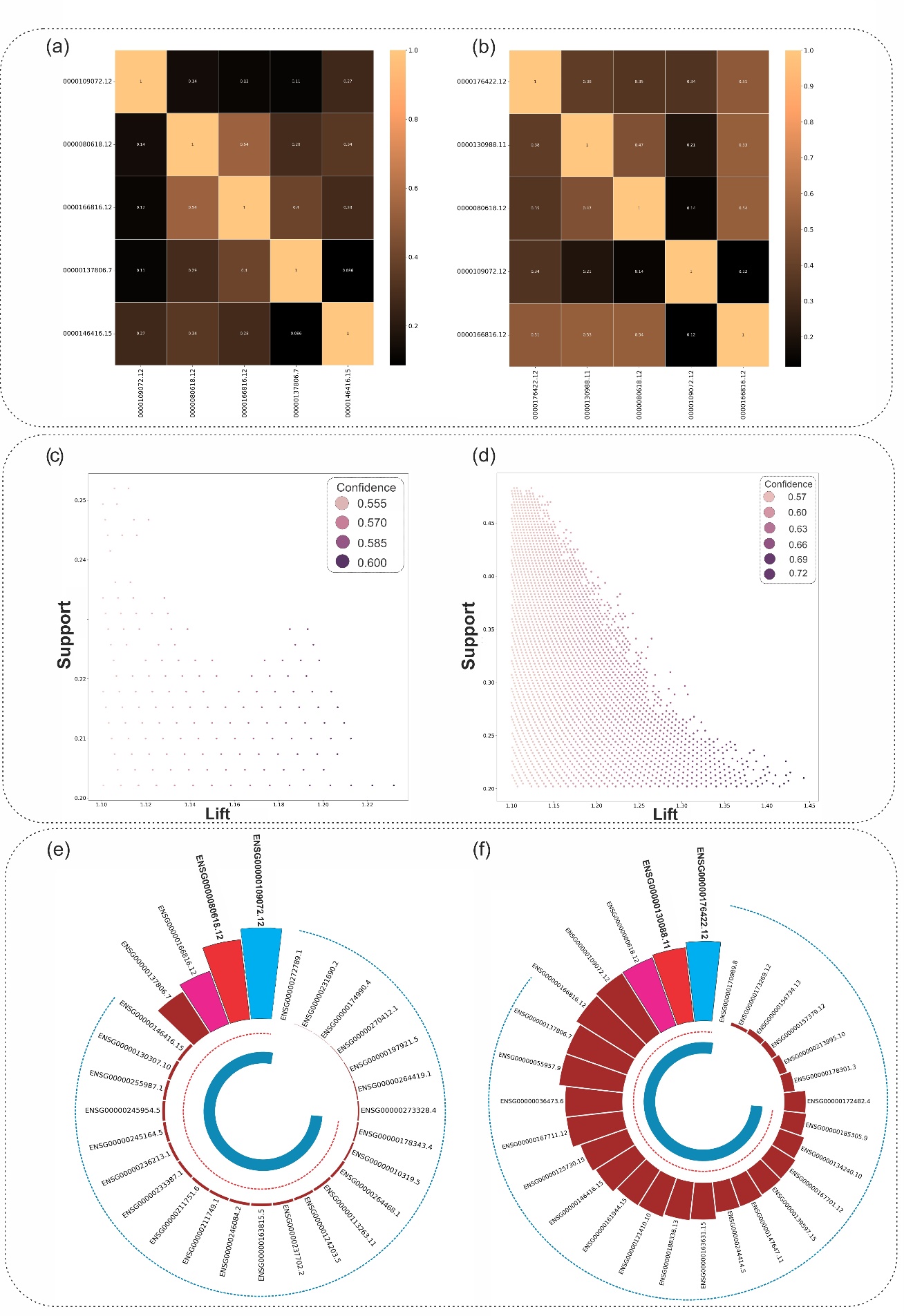


**Figure S3. Identified mRNAs at the early and late stages of HCC.** The heatmap plot based on the Spearman correlation for five top mRNAs of a) early-stage and b) late-stage rules. Strength distribution of c) early-stage and d) late-stage association rules according to their support, lift, and confidence. Ring bar plot of the repeat count of 28 top mRNAs in e) early-stage and f) late-stage rules. Python programming language (version 3.9) and Matplotlib library (version 3.6.0) were used to draw the heatmap plot, all of them are open sources.
